# Mesenchymal stem cell alleviate concanavalin A-induced hepatitis via immune reprogramming and complement regulation

**DOI:** 10.3389/fimmu.2026.1809194

**Published:** 2026-05-13

**Authors:** Hanpeng Luo, Bing Liu, Yaqin Li, Peng Cui, Tao Zhou, Cai Ye, Can Wu, Junchang Wu, Yu Wang, Wei V. Zheng

**Affiliations:** 1 Intervention and Cell Therapy Center, Peking University Shenzhen Hospital, Shenzhen, Guangdong, China; 2 Department of Infectious Disease, Peking University Shenzhen Hospital, Shenzhen, Guangdong, China; 3 Joint National Laboratory for Antibody Drug Engineering, School of Medicine, Henan University, Kaifeng, Henan, China; 4 Shenzhen 150 Life Technology Co., Ltd., Shenzhen, Guangdong, China; 5 Department of Clinical Laboratory Sciences, Yanjing Medical College, Capital Medical University, Beijing, China

**Keywords:** complement signaling, immune-mediated hepatitis, macrophage polarization, mesenchymal stem cells, single-cell RNA sequencing

## Abstract

**Background:**

Concanavalin A (ConA)-induced acute hepatitis is a widely used murine model for studying immune-mediated liver injury, characterized by T-cell activation and pro-inflammatory cytokine production. Mesenchymal stem cells (MSCs) have shown promise in mitigating liver injury through immunomodulation, but the precise cellular and molecular mechanisms remain unclear. This study leverages single-cell RNA sequencing (scRNA-seq) to elucidate the role of MSCs in reshaping the hepatic immune microenvironment during acute liver injury.

**Methods:**

Single-cell suspensions were isolated from liver tissues of ConA-induced acute hepatitis mice, with or without MSC treatment. ScRNA-seq libraries were generated using the 10× Genomics platform, and data were processed using Seurat for quality control, clustering, and cell-type annotation. Trajectory and pseudotime analysis were performed using Monocle 3 to model differentiation pathways. Ligand-receptor interactions were analyzed using CellChat to identify active signaling pathways. Key findings were further assessed *in situ* by multiplex immunohistochemistry (mIHC).

**Results:**

MSC administration markedly alleviated ConA-induced acute liver injury. scRNA-seq analysis showed that the global hepatic cellular landscape remained largely dominated by the acute inflammatory challenge, whereas MSC treatment was associated with selective remodeling of specific immune compartments. In particular, MSC treatment reduced the proportions of MDSCs, Tregs, NK cells, and proliferating CD8^+^ T cells, while increasing monocyte-derived macrophages (MoMFs). Subclustering and pseudotime analyses revealed heterogeneous MoMF states distributed along distinct transcriptional branches, including inflammatory and tissue-remodeling-associated programs. Combined with the compositional changes, these findings suggest selective remodeling of MoMF states following MSC treatment rather than uniform suppression of all macrophage subsets. Cell–cell communication analysis further identified MoMFs as a major signaling hub, with complement-associated signaling emerging as a prominent communication module directed toward downstream MDSCs and NK cells. Consistently, mIHC showed increased CD206-associated macrophage staining and reduced CD86 signal in MSC-treated livers compared with ConA-treated livers.

**Conclusion:**

MSCs ameliorate acute immune-mediated liver injury in association with selective remodeling of the hepatic immune microenvironment, particularly within the MoMF compartment. These findings support a model in which MSC treatment is linked to altered macrophage-state composition and MoMF-centered complement-associated communication during injury resolution, providing insight into the innate immune mechanisms underlying MSC-mediated hepatoprotection.

## 1 Introduction

Immune-mediated hepatitis represents a major pathological process underlying a wide spectrum of liver disorders, including autoimmune and viral hepatitis, characterized by extensive immune cell infiltration and cytokine-driven hepatocellular damage. The Concanavalin A (ConA)-induced hepatitis model is one of the most commonly used murine systems to study immune-mediated liver injury, recapitulating the pathophysiological and immunological features of human autoimmune hepatitis (1, 2). Following ConA administration, excessive activation of T cells, natural killer (NK) cells, and macrophages triggers massive secretion of proinflammatory cytokines such as tumor necrosis factor-α (TNF-α), interferon-γ (IFN-γ), and interleukin-6 (IL-6), which synergistically induce hepatocyte apoptosis and acute liver injury (3, 4). Although conventional immunosuppressive therapies, including corticosteroids and calcineurin inhibitors, can alleviate hepatic inflammation, their long-term application is often limited by systemic side effects, infection risk, and high recurrence rates (5). Therefore, novel therapeutic strategies that can restore immune homeostasis without global immune suppression are urgently needed.

Mesenchymal stem cells (MSCs) have emerged as a promising cell-based therapy for immune-mediated diseases owing to their potent immunomodulatory and regenerative properties. MSCs are generally believed to mediate tissue protection primarily through paracrine and immunomodulatory mechanisms, such as the secretion of soluble factors and extracellular vesicles, rather than through direct differentiation into functional liver cells or other tissue-resident cells (6, 7). Preclinical studies have demonstrated that MSCs ameliorate hepatic injury by reducing hepatocyte necrosis, lowering serum transaminase levels, and promoting tissue repair (8, 9). Mechanistically, MSCs suppress T-cell proliferation, induce regulatory T cells (Tregs), inhibit dendritic cell maturation, and promote macrophage polarization toward an anti-inflammatory M2 phenotype (10, 11). However, despite extensive research, the cellular and molecular mechanisms by which MSCs modulate the immune microenvironment in immune-mediated liver injury remain incompletely defined.

Recent advances in single-cell RNA sequencing (scRNA-seq) have revolutionized our understanding of immune responses by enabling the dissection of cellular heterogeneity and the reconstruction of intercellular signaling networks at single-cell resolution (12, 13). scRNA-seq provides an unbiased platform to identify rare immune subsets, delineate dynamic activation states, and reveal key ligand–receptor interactions within complex tissue environments. In the liver, single-cell transcriptomic analyses have uncovered distinct populations of macrophages, T cells, and endothelial cells with specialized functions in inflammation and regeneration (14, 15). However, how MSC therapy reshapes the hepatic immune landscape at single-cell resolution, particularly during acute immune activation, remains largely unknown.

In this study, we established a murine model of ConA-induced acute hepatitis and performed integrated histological, biochemical, and single-cell transcriptomic analyses to elucidate how MSCs modulate hepatic immune responses. By combining bulk biochemical assays with high-resolution scRNA-seq profiling, we provide a comprehensive cellular and molecular atlas of MSC-mediated protection. Our findings reveal that MSC treatment suppresses proinflammatory cytokine production, reprograms monocyte–macrophage polarization, attenuates cytotoxic T-cell activation, and reconstructs intercellular communication networks toward immune resolution and tissue repair. These results highlight the MSC–macrophage–complement axis as a pivotal pathway driving immunomodulation and regeneration in acute immune-mediated liver injury.

## 2 Materials and methods

### 2.1 Animal models and experimental design

Female BALB/c mice (8–10 weeks old, 18–20 g) were purchased from a certified specific pathogen–free (SPF) facility. All animals were acclimatized for one week under controlled environmental conditions (22 ± 2 °C, 50–60% humidity, 12 h light/dark cycle) with free access to food and water. After acclimation, the mice were randomly divided into four groups (n = 6 per group): 1) Control group: mice received no drug or cell treatment and were maintained under standard housing conditions. 2) ConA group: mice were injected via the tail vein with Concanavalin A (ConA; L7647, Sigma-Aldrich, USA) at a dose of 15 mg/kg, dissolved in sterile phosphate-buffered saline (PBS; 1 mg/mL). 3) MSC group: mice received an intravenous injection of 200 μL of MSC suspension containing 1 × 10^6^ cells via the tail vein. 4) MSC + ConA group: mice were first administered ConA (15 mg/kg, tail vein) as above, followed 2 hours later by a tail-vein injection of 200 μL MSC suspension containing 1 × 10^6^ cells.

Human umbilical cord blood-derived MSCs were used in this study. The samples were obtained with written informed consent under the approval of the Ethics Committee of Peking University Shenzhen Hospital (Approval No. 2023-017). Mononuclear cells were isolated from the umbilical cord blood by density-gradient centrifugation using Ficoll-Paque PLUS (Cytiva) and cultured in α-Minimum Essential Medium (α-MEM; Gibco) supplemented with 10% fetal bovine serum (FBS; Gibco) and 1% penicillin-streptomycin (Gibco) at 37 C in a humidified atmosphere containing 5% CO_2_. After 72 hours of incubation, non-adherent cells were removed by washing with phosphate-buffered saline (PBS), and the medium was changed every 3 days. The adherent fibroblast-like cells were further expanded, and cells at passages 3 to 5 were used for subsequent experiments. MSCs were characterized based on plastic adherence, spindle-shaped morphology, and surface-marker expression. For flow cytometric analysis, cells were harvested and stained with fluorochrome-conjugated antibodies against human CD73, CD90, CD105, CD34, CD45, and HLA-DR (BD Biosciences) in the dark at 4 C for 30 minutes.

At 24 hours after ConA injection, all mice were anesthetized, and blood samples were collected from the retro-orbital plexus (blood samples were collected via enucleation). Mice were then euthanized by cervical dislocation. Serum was obtained by centrifugation (3,000 rpm, 10 min) and stored at -80 C for subsequent biochemical and cytokine analyses. Whole liver tissues were harvested, photographed, and divided into portions for histopathological analysis, RNA extraction, and protein assays. Portions were fixed in 4% paraformaldehyde for hematoxylin-eosin (H&E) staining, while others were snap-frozen in liquid nitrogen and stored at −80 C for molecular assays. All procedures were repeated in triplicate to ensure experimental reproducibility, and all animal protocols were approved by the Institutional Animal Care and Use Committee of the Shenzhen PKU-HKUST Medical Center (Approval No. 2024-118).

### 2.2 Hematoxylin & eosin staining

Liver tissues were fixed in 4% paraformaldehyde for 24 hours, dehydrated through graded ethanol, embedded in paraffin, and sectioned at 4 μm thickness. Sections were deparaffinized in xylene, rehydrated with descending ethanol concentrations, and rinsed in distilled water. Slides were stained with hematoxylin for 5–10 min, differentiated in 1% acid alcohol, blued in alkaline solution, and counterstained with eosin for 1–3 min. After dehydration through graded ethanol and clearing in xylene, slides were mounted with neutral resin. Histopathological changes, including hepatocellular necrosis, inflammatory cell infiltration, and structural alterations, were examined under a light microscope (Leica Microsystems, Germany) and photographed for analysis.

### 2.3 TUNEL assay for hepatocyte apoptosis

Hepatocyte apoptosis was evaluated using a terminal deoxynucleotidyl transferase dUTP nick-end labeling (TUNEL) assay kit (Roche, Germany). Paraffin-embedded liver sections were deparaffinized, rehydrated, and treated with proteinase K (20 μg/mL) for 20 min at 37 C to expose DNA ends. After washing with PBS, sections were fixed in 4% paraformaldehyde for 30 min and permeabilized with 0.1% Triton X-100 on ice for 10 min. Slides were then incubated with the TUNEL reaction mixture containing TdT enzyme and fluorescein-labeled dUTP for 1 h at 37 C in the dark. After washing, nuclei were counterstained with DAPI (1 μg/mL) for 5 min, mounted with anti-fade medium, and observed under a fluorescence microscope (Leica Microsystems, Germany). Apoptotic cells displaying green fluorescence were counted in randomly selected microscopic fields, and the apoptotic index was calculated as the percentage of TUNEL-positive nuclei relative to total hepatocyte nuclei.

### 2.4 Serum biochemical indicators and cytokine

At 24 hours after ConA injection, blood samples were collected from the retro-orbital plexus and centrifuged at 3,000 rpm for 10 minutes to obtain serum. All serum samples were stored at −80 C until analysis. Serum biochemical indices, including alanine aminotransferase (ALT), aspartate aminotransferase (AST), alkaline phosphatase (ALP), total bile acids (TBA), total bilirubin (T-BIL), direct bilirubin (D-BIL), and indirect bilirubin (I-BIL), were determined using commercial assay kits (melkang, China) according to the manufacturer’s instructions. The enzymatic activities or absorbance changes at the corresponding wavelengths were automatically recorded using a fully automated biochemical analyzer to evaluate hepatic injury and liver function. Serum cytokine concentrations were measured using a multiplex bead-based Luminex assay (ProcartaPlex™, Thermo Fisher Scientific, USA) or enzyme-linked immunosorbent assay (ELISA).

A total of 36 cytokines and chemokines were quantified in mouse serum. Serum cytokine levels were measured using the ProcartaPlex Mouse Cytokine/Chemokine Panel 1A 36plex kit (Invitrogen, EPX360-26092-901) according to the manufacturer’s protocol. The assay was independently conducted and analyzed by Laizee Biotech (Shanghai, China) using a Luminex 200 system and ProcartaPlex Analyst 1.0 software. Briefly, 25 μL of each serum sample was incubated for 2 hours at room temperature with a mixture of color-coded magnetic beads pre-coated with analyte-specific capture antibodies. After washing, biotinylated detection antibodies and streptavidin–phycoerythrin (SA–PE) were sequentially added to form the antibody–antigen–antibody complexes. The beads were analyzed on a Luminex 200 instrument, where one laser identified each bead set and a second laser quantified the PE signal intensity proportional to the analyte concentration. Standard curves for all 36 analytes were generated, and concentrations were calculated using a five-parameter logistic (5-PL) regression model. Cytokine concentrations were expressed as pg/mL, and all assays were performed in duplicate. The mean values were used for statistical analysis.

### 2.5 Single-cell RNA sequencing

Single-cell suspensions were generated from fresh liver tissues. Briefly, liver tissues were minced into small pieces and digested enzymatically with collagenase IV and DNase I at 37 C. The digested tissues were filtered through a 70-μm cell strainer, and red blood cells were removed by lysis buffer treatment. After washing, viable cells were resuspended in PBS containing 0.04% BSA and used for downstream single-cell library construction. ScRNA-seq libraries were prepared using the 10× Genomics Chromium platform following the manufacturer’s protocols. Sequencing was conducted on the Illumina NovaSeq 6000 platform. Raw sequencing reads underwent alignment and processing with Cell Ranger (v7.2). We identified and removed doublets using DoubletFinder (16) (v2.0.4) and corrected for ambient RNA contamination using decontX (17) (v1.0.0). Cells were further filtered based on gene detection and library complexity, retaining only those with intermediate gene counts (200–6,000 genes detected), estimated contamination fractions <0.9 (less than 90% ambient RNA), and log10-transformed genes-per-UMI ratios greater than 0.8. Downstream analyses including quality control, clustering, and annotation were performed using Seurat v5.3.

### 2.6 Cell-type identification and immune diversity

Sample integration was performed using the Harmony integration method to correct for batch effects across different samples. A nearest-neighbor graph was constructed using the top 30 principal components. Clustering was performed on the graph using a resolution of 0.6 to identify initial clusters based on gene expression profiles. Cell-type annotation was guided by known marker genes. Differential gene expression was performed using the Wilcoxon rank-sum test with adjusted p-values (Benjamini-Hochberg method). Shannon entropy was calculated to quantify immune diversity. Principal component analysis (PCA) was used to assess overall cell-type distribution differences among groups.

### 2.7 Pseudotime analysis and functional enrichment

To explore the differentiation trajectories of macrophage subpopulations and their response to MSC treatment, Monocle 3 (18) was used to perform pseudotime analysis. This approach allowed the cells to be ordered along a differentiation trajectory based on their gene expression profiles. Branch heatmaps and scatterplots were used to visualize gene expression dynamics along pseudotime, revealing the temporal progression of macrophage differentiation and polarization during liver inflammation and repair. Functional enrichment analyses were conducted using clusterProfiler (19).

### 2.8 Ligand-receptor interactions and pathway analysis

Cell-cell communication was analyzed using CellChat (20), an R package that identifies ligand-receptor interactions between different cell populations. This analysis allowed us to explore the active signaling pathways involved in immune modulation, particularly the interactions between Kupffer-like macrophages (MoMFs), Tregs, NK cells, and dendritic cells. We specifically focused on complement-related signaling, visualizing the expression of key molecules using violin plots to assess pathway activation and cell–cell interaction dynamics.

### 2.9 Multiplex immunohistochemistry and multichannel imaging

Paraffin-embedded liver tissue sections were deparaffinized in xylene and rehydrated through a graded ethanol series. Antigen retrieval was performed using AR buffer (Panovue Biosciences) in a microwave oven, followed by quenching of endogenous peroxidase activity with 3% hydrogen peroxide for 10 min. Multiplex immunohistochemistry (mIHC) staining was conducted through multiple iterative cycles. Each cycle consisted of a blocking step with 1% bovine serum albumin (BSA), incubation with the primary antibody, and subsequent incubation with the corresponding horseradish peroxidase (HRP)-conjugated secondary antibody (Panovue Biosciences). Tyramide signal amplification (TSA) was performed using different TSA-conjugated chromogens (1:100 in TSA diluent, Panovue Biosciences). After each round of staining, the covalent linkage of the TSA chromogen to the target epitope was completed, and the bound antibodies were removed by repeating the antigen retrieval step to enable the next staining cycle. The sequence of primary antibodies and TSA chromogens was as follows: anti-CD11c (ab219799, 1:100, Abcam)/TSA-PPD 620, anti-CD206 (24595S, 1:1000, Cell Signaling Technology)/TSA-PPD 520, anti-F4/80 (70076S, 1:500, Cell Signaling Technology)/TSA-PPD 690, and anti-CD86 (19589S, 1:800, Cell Signaling Technology)/TSA-PPD 570. After completion of all staining rounds, the sections were counterstained with spectral DAPI (Akoya Biosciences) and mounted with anti-fade fluorescence mounting medium (ab104135, Abcam). Multichannel bright-field imaging was acquired using a PANNORAMIC SCAN II imaging system (3DHISTECH, Hungary) at ×200 magnification.

## 3 Results

### 3.1 MSCs alleviates ConA -induced acute liver injury

**Figure 1:**
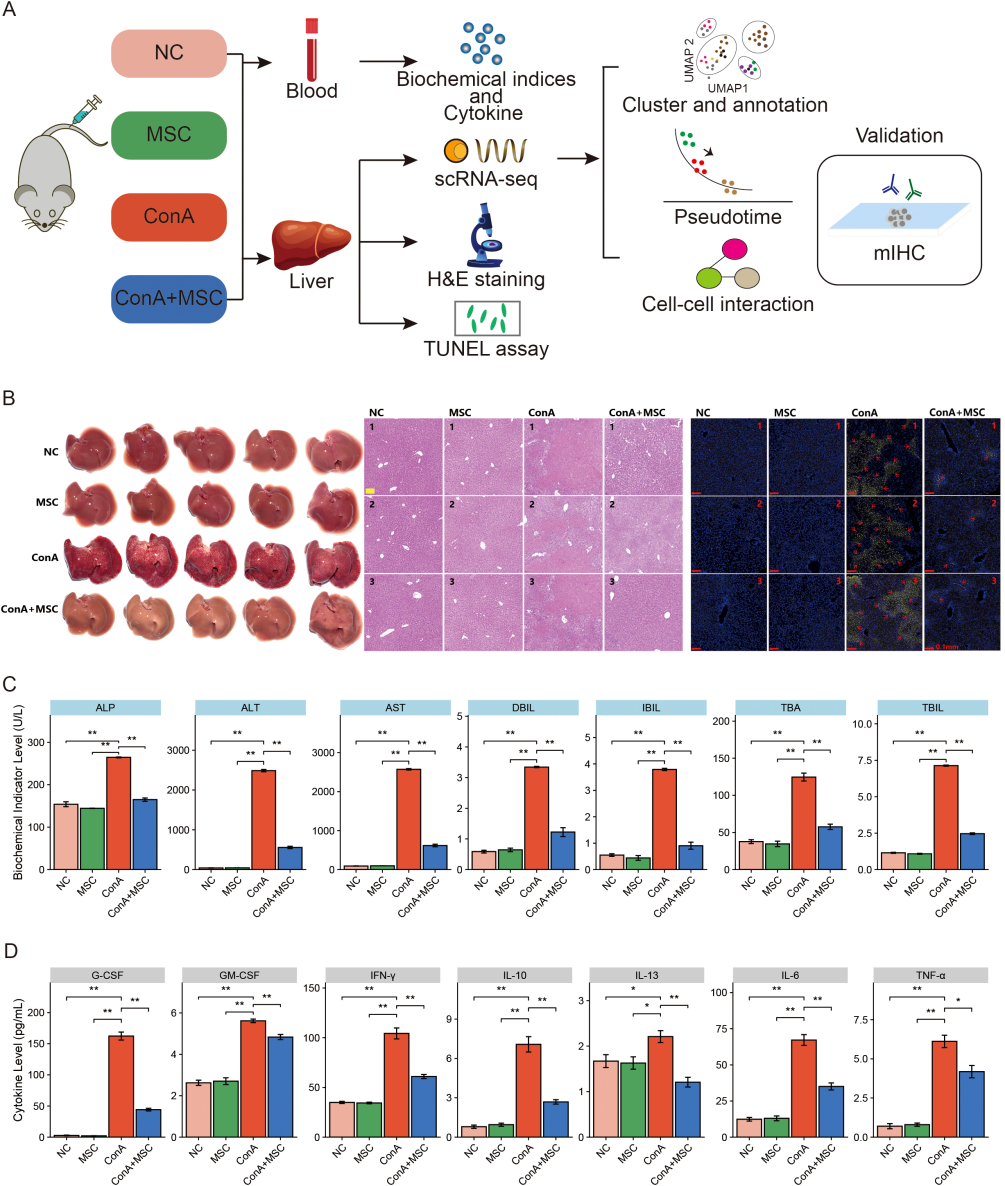
Experimental design, tissue morphology, and biochemical analysis. **(A)** Experimental workflow: Mice were assigned to four treatment groups (NC, MSC, ConA, ConA+MSC). Blood and liver samples were collected for biochemical, scRNA-seq, H&E, and TUNEL assays. **(B)** Histological analysis of liver tissue. Left: Macroscopic liver images showing differences between treatment groups. Middle: H&E staining of liver sections showing cellular infiltration in the ConA group and improved histology in the ConA+MSC group. Right: TUNEL assay showing apoptotic cells in the ConA group. **(C)** Serum biochemical indices of liver injury. The elevated levels of hepatic markers (ALP, ALT, AST, DBIL, IBIL, TBA, and TBIL) induced by ConA were significantly attenuated following MSC administration (ConA+MSC group). **(D)** Plasma cytokine levels. ConA challenge triggered a robust release of systemic cytokines (including G-CSF, GM-CSF, IFN-γ, IL-10, IL-13, IL-6, and TNF-α), whereas MSC treatment (ConA+MSC group) significantly suppressed this cytokine storm. *p < 0.05, **p < 0.01.

We established an experimental model of acute liver injury using ConA in mice and evaluated the effects of MSCs on liver protection. Four experimental groups were included: NC (Control), MSC, ConA, and ConA+MSC. The experimental design is illustrated in **Figure 1A**, which summarizes the treatment groups and the series of analyses performed, including biochemical indices, cytokine, scRNA-seq, histology (H&E), and TUNEL assay.

The macroscopic examination of liver tissues in **Figure 1B** (left panel) shows dark red and markedly congested livers in the ConA group compared to other groups, indicating the presence of liver damage. Histopathological analysis via H&E staining (**Figure 1B** middle panel) further reveals substantial hepatocyte injury and inflammatory cell infiltration in the ConA-treated group, whereas the ConA+MSC groups displayed less damage. In TUNEL assay (**Figure 1B** right panel), we observed high levels of hepatocytes apoptosis in the ConA group, while the ConA+MSC group exhibited reduced apoptosis, supporting the protective role of MSCs.

We analyzed biochemical markers associated with liver injury in **Figure 1C**, including ALP, ALT, AST, DBIL, IBIL, TBA, and TBIL. Consistent with the observed histological damage, liver injury markers were significantly elevated in the ConA-treated group, indicating extensive hepatocyte damage. In contrast, the MSC-alone group showed no significant difference compared with the normal control (NC), indicating that MSC administration itself caused no hepatic toxicity. Remarkably, the ConA + MSC group displayed substantial restoration of these biochemical indices, demonstrating the hepatoprotective effects of MSC therapy. We next assessed cytokine levels in plasma as an indicator of immune activation (**Figure 1D****;** **Supplementary Figure 1**). Elevated levels of various cytokines, including pro-inflammatory markers such as TNF-α, IL-6, and IFN-γ, were detected in the ConA-treated group compared to the NC and MSC groups. Importantly, the administration of MSCs (ConA+MSC group) significantly reduced the levels of all these ConA-induced cytokines, including IL-10. These findings suggest that MSCs exert a potent anti-inflammatory effect, effectively dampening the overall cytokine storm induced by ConA.

### 3.2 MSC treatment remodels hepatic immune composition in ConA-induced liver injury

**Figure 2:**
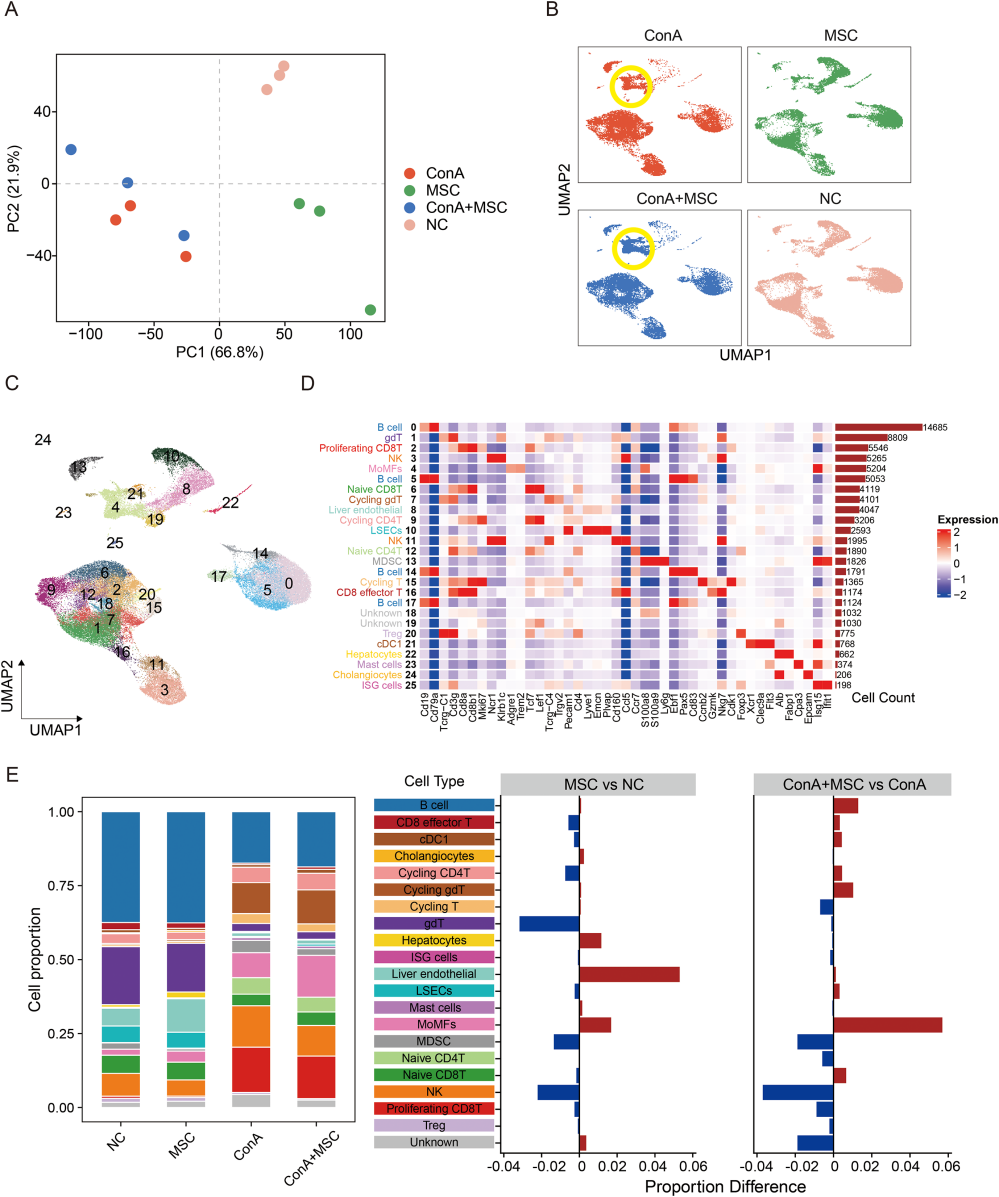
Single-cell profiling of hepatic immune cells in ConA-induced liver injury with MSC treatment. **(A)** PCA of immune cell composition across groups (NC, MSC, ConA, ConA+MSC), highlighting clear separation based on treatment conditions. **(B)** UMAP plots demonstrating cell distribution for each experimental condition. **(C)** UMAP projection identifies 26 distinct liver cell populations from integrated scRNA-seq data. **(D)** Heatmap showing the expression patterns of canonical marker genes across identified cell types, with cell counts depicted on the right. **(E)** Relative cell proportions in each condition (left), with proportion differences comparing MSC vs NC and ConA+MSC vs ConA (right).

To further investigate the immunomodulatory mechanisms of MSCs in ConA-induced acute hepatitis, we performed scRNA-seq on liver tissues from all experimental groups (NC, MSC, ConA, and ConA+MSC). After quality control and filtering (**Supplementary Figures 2A, B**), a total of 78,838 high-quality single cells were retained for downstream analysis. PCA showed that the major global transcriptional variation was primarily driven by ConA treatment, separating the homeostatic groups (NC and MSC) from the acute injury groups (ConA and ConA+MSC) (**Figure 2A**). The close clustering of the ConA and ConA+MSC samples suggests that ConA-induced acute inflammation dominated the overall transcriptomic landscape. Consistently, batch-corrected UMAP analysis showed that although the global cellular architecture remained largely shaped by acute injury, MSC treatment induced selective shifts within specific immune compartments rather than a full return to homeostasis (**Figure 2B****;** **Supplementary Figure 2C**). Integrated analysis identified 26 transcriptionally distinct clusters representing major hepatic and immune cell populations, including hepatocytes, LSECs, MoMFs, B cells, CD4^+^ and CD8^+^ T cells, NK cells, MDSCs, and Tregs (**Figures 2C, D**).

Cell-type proportion showed that the ConA challenge was the main driver of cellular remodeling (**Figure 2E**). Compared with controls, the ConA group displayed expansion of injury-associated immune populations, including cycling CD8^+^ T cells, NK cells, and MDSCs, together with reduced hepatocytes and LSECs. MSC treatment partially reshaped this immune landscape, with decreased NK cells, proliferating CD8^+^ T cells, and MDSCs, while MoMFs were increased, suggesting selective remodeling of the hepatic myeloid compartment during injury resolution.

Notably, MoMFs in the MSC-treated group displayed downregulation of pro-inflammatory markers (*S100a8*, *S100a9*, *Il1b*) and upregulation of immune-modulatory and tissue-repair genes (*Apoe*, *C1qa*, *Trem2*), suggesting a functional shift toward a regulatory macrophage phenotype (**Supplementary Table 1**). Together, these findings indicate that MSC therapy alleviates ConA-induced hepatic inflammation by restoring cellular heterogeneity and promoting immune tolerance within the liver microenvironment.

### 3.3 Functional characterization reveals specialization among MoMF subpopulations

**Figure 3:**
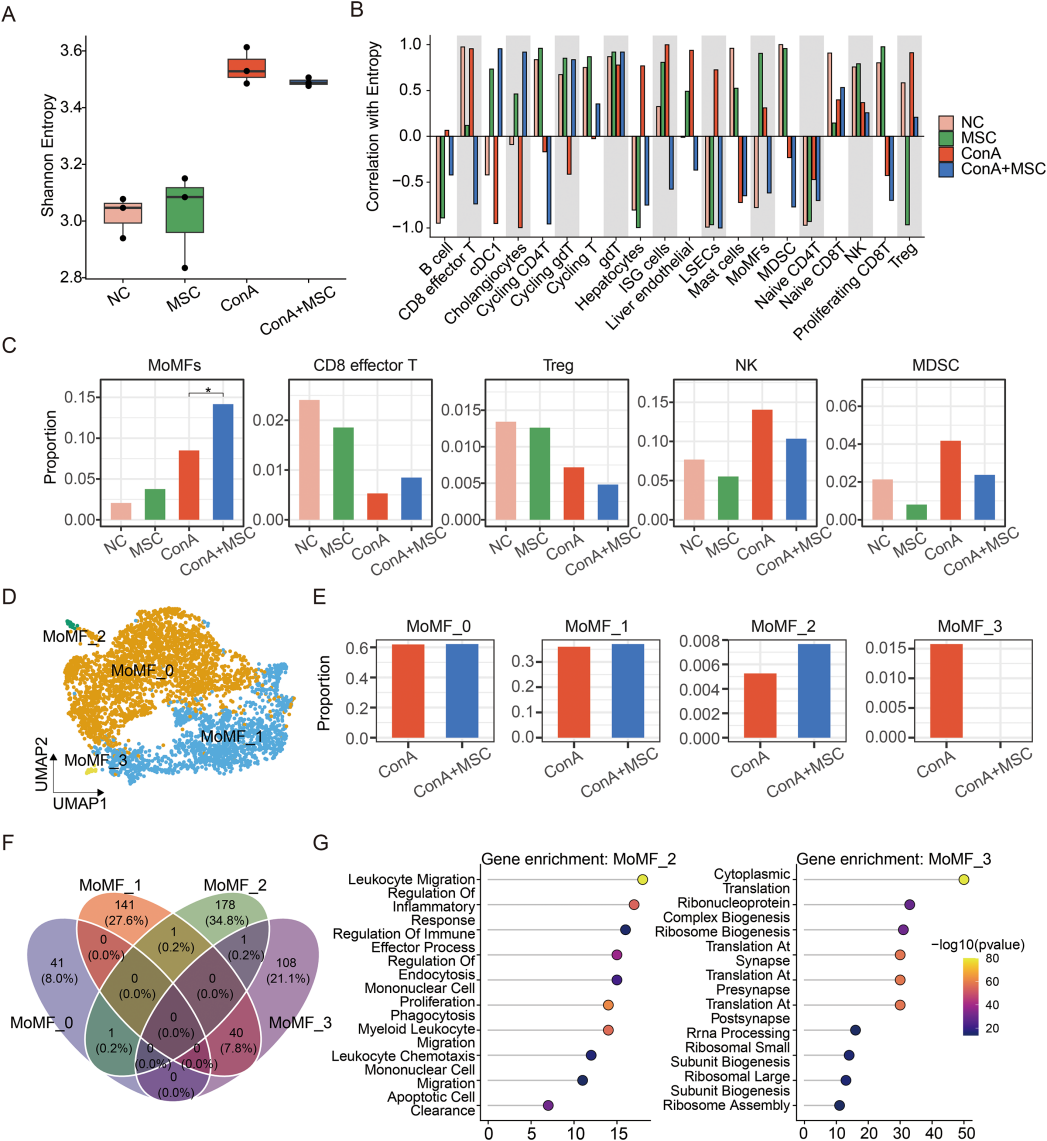
MSC treatment restores immune diversity and modulates monocyte-derived macrophage (MoMF) subsets. **(A)** Shannon entropy analysis of immune diversity across treatment groups. **(B)** Correlation of immune cell proportions with Shannon entropy across groups, indicating cell types contributing to immune diversity. **(C)** Differential analysis of selected immune cell populations (MoMFs, CD8 effector T cells, Tregs, NK cells, MDSCs) among experimental groups (*p < 0.05). **(D)** UMAP visualization of MoMF subtypes (MoMF_0–3). **(E)** Relative proportions of MoMF subtypes between ConA and T+ConA groups. **(F)** Venn diagram showing unique and shared genes among MoMF subsets. **(G)** Functional enrichment of MoMF_2 and MoMF_3 subtype-specific genes, highlighting immune-related pathways.

To further dissect the immunoregulatory mechanisms of MSCs in ConA-induced hepatitis, we evaluated the overall immune heterogeneity and macrophage subpopulation dynamics among groups using Shannon entropy analysis and subset profiling. Shannon entropy analysis demonstrated that global immune diversity was markedly increased in the ConA group compared to physiological controls (**Figure 3A**). Following MSC treatment, this elevated level of diversity was maintained. Correlation analysis between cell-type abundance and entropy (**Figure 3B**) revealed that Tregs positively correlated with overall immune diversity, whereas MDSCs exhibited negative correlations in both injury and treatment states. Notably, MoMFs showed a positive correlation in the ConA group but a negative correlation in the ConA+MSC group. This indicates that MSC administration induces a profound qualitative reorganization of the hepatic immune microenvironment. The proportion of immune cells revealed striking differences among groups (**Figure 3C**). MoMFs were markedly expanded in the ConA + MSC group, suggesting that MSC therapy promotes the recruitment or differentiation of monocyte-derived macrophages involved in immune resolution. In contrast, CD8^+^ effector T cells were substantially reduced in the ConA group, indicating impaired cytotoxic activity during acute immune-mediated injury. Moreover, the proportions of NK cells and MDSCs were elevated in ConA-treated mice, the proportion of Tregs was decreased. Following MSC treatment, the elevated proportions of NK cells and MDSCs declined toward their respective baseline levels, whereas Tregs remained at lower levels. To better characterize the heterogeneity within MoMFs, subclustering analysis identified four transcriptionally distinct MoMF subsets (MoMF_0-3) (**Figure 3D**). These subclusters displayed distinct gene expression signatures and functional roles. Subset proportion (**Figures 3E, F**) revealed divergent dynamic shifts among these subclusters following treatment. Notably, the MoMF_2 subset, whose proportion was increased following MSC administration, was heavily enriched for classic scavenger receptors and tissue-repair genes (**Supplementary Table 2**), including *Marco*, *Apoe*, *C1qa*, *Mertk*, and *Cd163*. In contrast, the MoMF_1 subset expressed high levels of classical myeloid inflammatory markers, such as *S100a8* and *S100a9*. Furthermore, the MoMF_3 subset, which emerged specifically in the ConA-injured liver but was virtually eradicated following MSC administration, displayed a unique signature characterized by a massive upregulation of ribosomal genes (*Rps2*, *Rpl5*), alarmins (*Hmgb1*), and cytotoxic-associated markers (*Gzmb*). Gene Ontology (GO) enrichment analysis of these subsets revealed distinct biological processes, with significant enrichment in pathways associated with phagocytic clearance and immune cell recruitment (**Figure 3G**). Taken together, these data demonstrate that MSC administration profoundly alters the hepatic immune landscape by actively expanding highly specialized scavenger macrophage populations while eliminating hyper-active, stress-associated subsets.

### 3.4 MSC treatment directs MoMF differentiation trajectories

**Figure 4:**
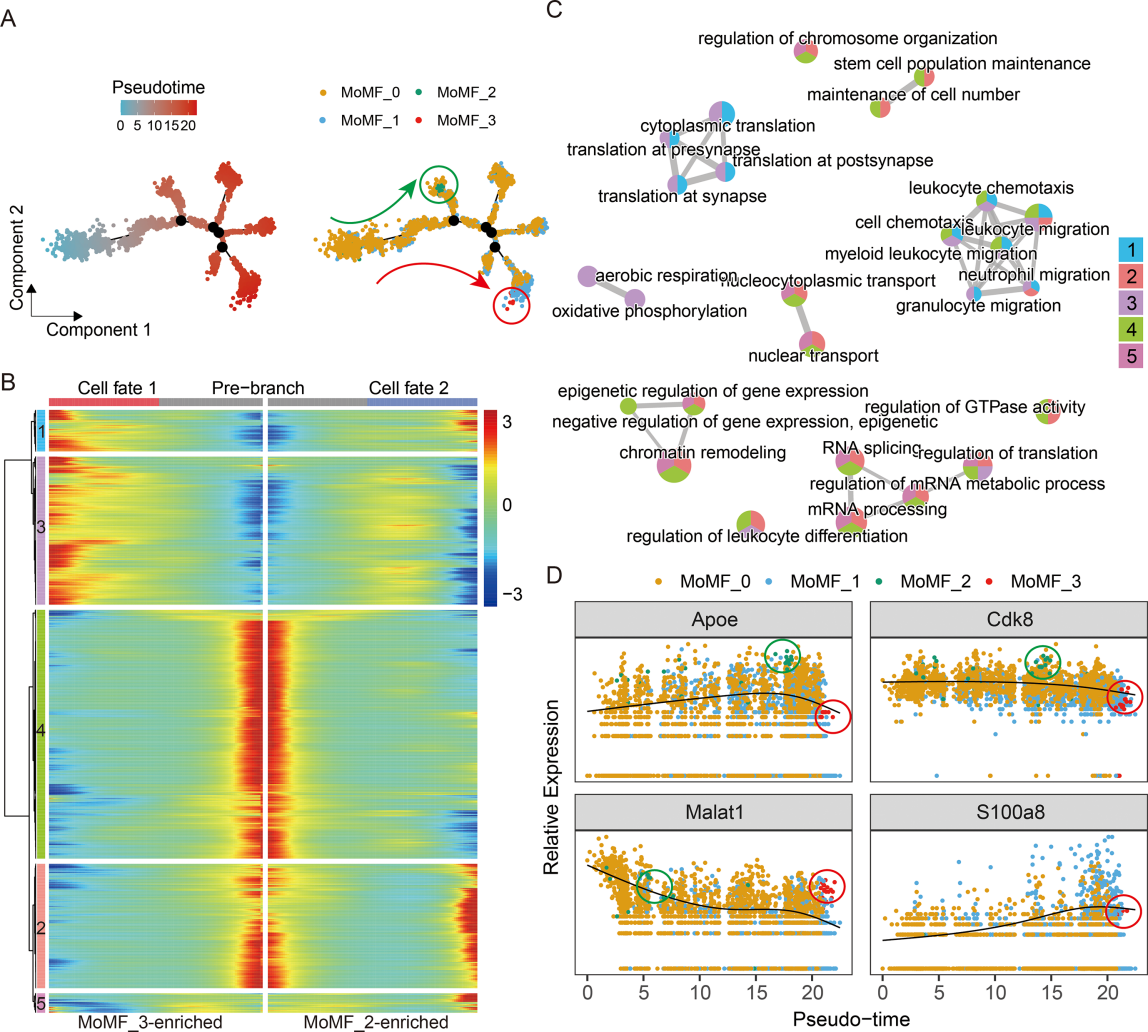
Trajectory analysis reveals distinct differentiation pathways in MoMF subsets. **(A)** Pseudotime trajectory of MoMF differentiation, colored by pseudotime progression (left) and MoMF subtypes (right). **(B)** Heatmap of dynamically expressed genes along the pseudotime trajectory, categorized into pre-branch and two divergent cell fates. **(C)** Network plot illustrating enriched biological processes during MoMF differentiation, grouped by functional modules. **(D)** Expression dynamics of selected genes (Apoe, Cdk8, Malat1, S100a8) along pseudotime trajectory, highlighting distinct expression trends in MoMF subtypes.

To characterize transcriptional state transitions within MoMFs, pseudotime trajectory analysis was performed. The inferred trajectory suggested that MoMFs were distributed along divergent transcriptional branches originating from a shared early state (**Figure 4A**). towardMoMF_3 showed clear enrichment at one terminal branch, whereas MoMF_2 displayed relative enrichment along alternative branch trajectories, indicating branch-associated heterogeneity. To further interpret these branch-dependent programs, we analyzed gene-expression dynamics across the branching structure (**Figure 4B**) and performed functional annotation of branch-associated gene modules (**Figure 4C**). These analyses revealed distinct transcriptional programs across the trajectory. In particular, the MoMF_3-enriched terminal branch (Cell fate 1) was linked to gene modules related to translation, ribosome biogenesis, and oxidative phosphorylation, whereas the alternative branches with relative enrichment of MoMF_2 (Cell fate 2) were associated with programs related to cellular reprogramming. Analysis of representative marker genes along pseudotime further highlighted branch-specific transcriptional divergence (**Figure 4D**). S100a8 expression remained relatively stable across most of the trajectory but increased sharply at the terminal branch enriched for MoMF_3 cells. Malat1 showed an overall declining trend along pseudotime, yet displayed a local increase at the same branch. In contrast, Apoe remained broadly expressed across alternative branches. Together, these results suggest that infiltrating MoMFs occupy heterogeneous transcriptional states along divergent pseudotime branches, with the MoMF_3-associated branch marked by a distinct late inflammatory/stress-associated transcriptional program.

### 3.5 Complement signaling mediates MSC-induced immunomodulation via MoMFs

**Figure 5:**
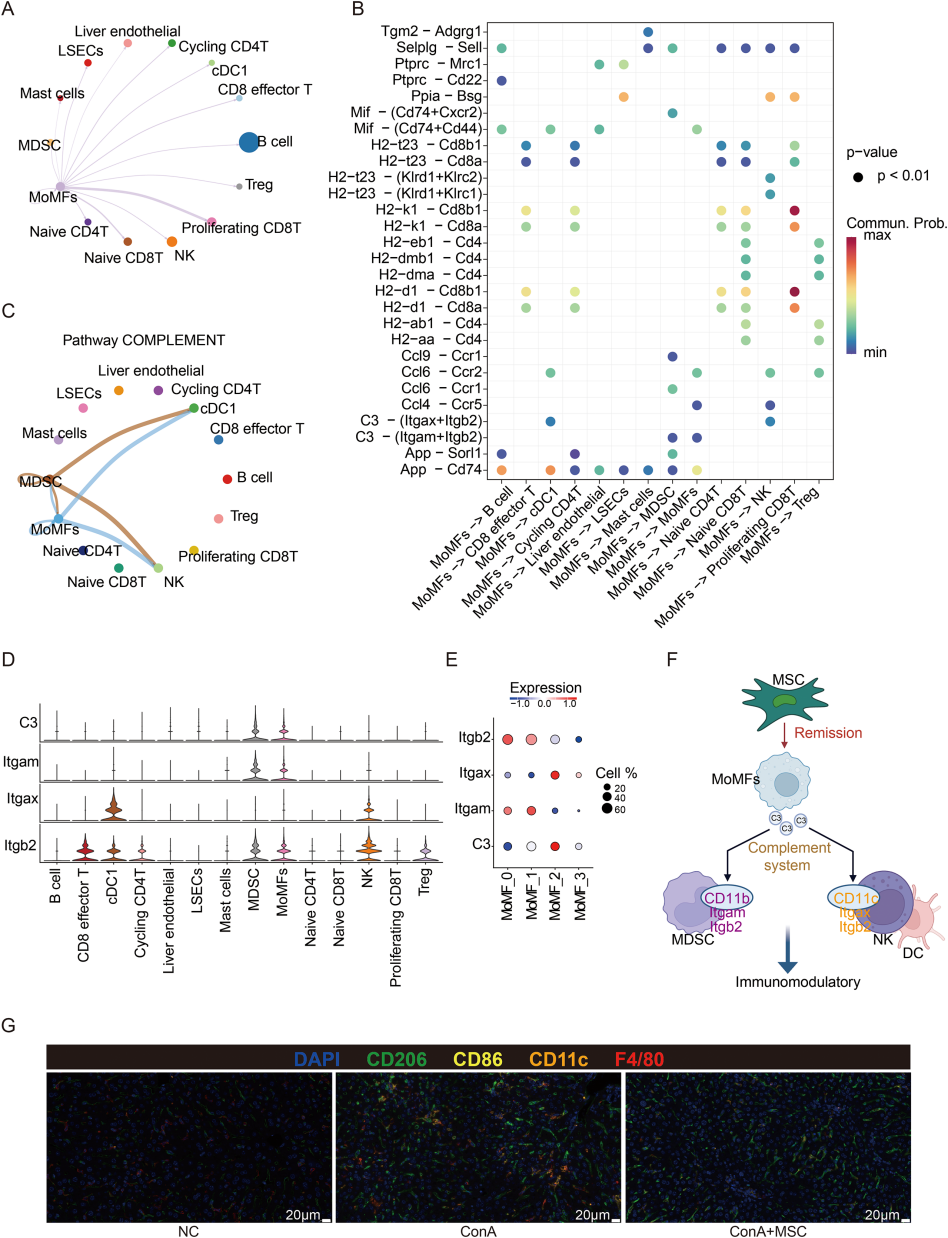
MSC activates complement-mediated immunomodulation via MoMFs. **(A)** Ligand-receptor interactions originating from MoMFs towards various immune populations, as predicted by CellChat. **(B)** Dot plot illustrating significant ligand-receptor pairs involved in MoMF communication with immune cell subsets. **(C)** Visualization of complement signaling pathways mediated by MoMFs interacting with immune cells. **(D)** Violin plots depicting expression levels of complement-related genes (C3, Itgam, Itgax, Itgb2) across hepatic immune cell subsets. **(E)** Violin plots depicting expression levels of complement-related genes (C3, Itgam, Itgax, Itgb2) across hepatic MoMF subsets. **(F)** Schematic representation summarizing MSC-mediated activation of the complement system through MoMFs, subsequently regulating immunomodulation via MDSC, NK, and dendritic cells. **(G)** Representative multiplex immunohistochemistry (mIHC) images showing CD206^+^, CD86^+^, CD11c^+^, and F4/80^+^ macrophages in liver tissues from NC, ConA, and ConA+MSC groups (scale bar = 20 μm).

To characterize the intercellular communication landscape associated with MoMFs, we performed cell-cell interaction analysis based on the scRNA-seq data. Network inference suggested that MoMFs constitute a major signaling hub within the hepatic microenvironment, displaying extensive interactions with multiple immune populations, including B cells, NK cells, and T-cell subsets (**Figure 5A**). Analysis of ligand-receptor pairs originating from MoMFs identified diverse candidate signaling interactions (**Figure 5B**). Among these, pathway-level network analysis highlighted complement-related signaling as a prominent communication module (**Figure 5C**). In this network, MoMFs appeared to be a major sender of complement-associated signals, with predicted interactions involving MDSCs, NK cells, and cDC1 cells. Expression profiling of the corresponding ligands and receptors across cell populations (**Figure 5D**). Specifically, C3 expression was enriched in MoMF_2 (**Figure 5E**) and MDSCs. Given that MSC administration selectively promotes the expansion of the MoMF_2 population, these data suggest that MSC treatment augments local complement signaling capacity via macrophage subset remodeling. Together, these data suggest that MoMF-centered complement-associated signaling may contribute to immune remodeling in the injured liver (**Figure 5F**).

To further assess macrophage-associated phenotypes at the tissue level, we performed multiplex immunohistochemistry (mIHC) on liver sections (**Figure 5G**). Compared with NC livers, ConA-treated livers showed marked infiltration of F4/80^+^ macrophages with increased CD86 and CD11c signals. In contrast, the ConA+MSC group showed stronger CD206-associated staining together with reduced CD86 signal relative to the ConA group. These findings are consistent with a shift in macrophage-associated marker expression after MSC treatment and support the transcriptomic observation that MSC administration is associated with remodeling of hepatic macrophage states.

## 4 Discussion

In the present study, we demonstrate that MSC therapy markedly alleviates ConA-induced acute hepatitis by suppressing hepatic inflammation, restoring immune homeostasis, and remodeling the hepatic immune microenvironment. Histopathological and biochemical analyses showed that MSC administration reduced hepatocellular necrosis and serum aminotransferase levels, consistent with previous reports demonstrating the hepatoprotective effects of MSCs in experimental liver injury models (6, 21, 22). Human MSCs were used in this murine model because MSC-mediated hepatoprotection is generally thought to rely mainly on conserved paracrine and immunomodulatory mechanisms rather than long-term engraftment. In this study, the xenogeneic model provides a preclinical system for examining the immunoregulatory effects of clinically relevant human MSCs. Using scRNA-seq, we constructed a comprehensive cellular and molecular atlas of hepatic immune dynamics, revealing that MSC treatment not only attenuated inflammatory immune activation but also reprogrammed macrophage and complement signaling to promote immune tolerance and tissue repair.

The ConA-induced hepatitis model mimics the immunopathology of autoimmune hepatitis through T cell-driven cytokine storm and extensive infiltration of inflammatory macrophages (2, 4). Our data confirm that MSC treatment substantially reduces ConA-triggered immune activation and normalizes inflammatory cytokines including TNF-α, IFN-γ, and IL-6. These findings are consistent with prior evidence that MSCs ameliorate hepatic injury by suppressing effector T-cell proliferation and inducing Tregs (1, 7, 10). MSCs have been shown to inhibit Th1/Th17 differentiation, promote IL-10 secretion, and restore the balance between Tregs and effector lymphocytes in autoimmune and viral hepatitis models (5, 23, 24). However, our high-resolution scRNA-seq data present a paradigm shift. Although MSC treatment substantially attenuated the expansion of cytotoxic effectors such as NK cells and normalized inflammatory cytokine networks, the proportion of Tregs remained at baseline or lower levels. This finding suggests MSCs do not strictly rely on the slow classical Treg-driven adaptive tolerance during hyper-acute hepatic inflammation. Instead, they rapidly establish an immunoregulatory microenvironment driven largely by massive myeloid and MDSC rewiring to prevent immediate and excessive tissue damage. Our serum cytokine profiling revealed that IL-10 was elevated in the ConA group but decreased after MSC treatment. One possible explanation is that, in the setting of hyper-acute inflammation, increased circulating IL-10 may represent a compensatory regulatory response to severe immune activation driven by cytokines such as TNF-α, IFN-γ, and IL-6. In this context, the subsequent decline in serum IL-10 after MSC treatment may be associated with attenuation of the upstream inflammatory burden rather than a loss of immune regulation. However, this interpretation remains tentative, and serum cytokine levels may not necessarily reflect local cytokine concentrations or activity within the hepatic microenvironment. A similar consideration applies to the reduced proportion of MDSCs after MSC treatment. Although MDSCs are generally regarded as immunosuppressive cells, their expansion in the ConA group may also reflect a compensatory regulatory response to severe inflammation, whereas their decline after MSC treatment may be consistent with reduced inflammatory stress.

Macrophages are central regulators of hepatic immunity and tissue repair (14, 15). In the setting of ConA-induced liver injury, our single-cell analysis identified multiple transcriptionally distinct MoMF states, highlighting dynamic macrophage diversification during acute inflammation. Among these, one branch was characterized by the robust expression of inflammatory genes, consistent with a pathogenic macrophage program associated with hepatocyte injury (25). In contrast, alternative MoMF states were enriched for transcriptional signatures related to phagocytosis, antigen handling, and tissue remodeling, suggesting reparative or resolution-associated features. Importantly, MSC treatment was not associated with a uniform suppression of all macrophage populations, but rather with selective remodeling of MoMF-state composition and transcriptional programs. Pseudotime analysis further suggested that infiltrating MoMFs were distributed along divergent transcriptional branches. One terminal branch showed marked enrichment of inflammatory features, whereas alternative branches retained broader expression of genes associated with phagocytic and tissue-remodeling functions. When interpreted together with the compositional changes observed after MSC treatment, these data support a model in which MSCs reshape macrophage-state transitions during liver injury resolution rather than simply inducing a binary shift in macrophage polarization. This interpretation is consistent with the established plasticity of macrophages and with previous studies showing that MSC-derived paracrine mediators can effectively modulate macrophage activation states and promote repair-associated phenotypes (26–28). Functionally, branch-associated gene modules in our dataset were linked to distinct metabolic, migratory, and leukocyte-trafficking programs, indicating that macrophage-state remodeling after MSC treatment involves coordinated changes in biosynthetic activity and tissue-response functions. Such remodeling is essential for apoptotic cell clearance, regulation of local inflammation, and support of tissue repair, which aligns with previous reports that reparative macrophage populations contribute to hepatocyte recovery, extracellular matrix remodeling, and the suppression of excessive inflammatory signaling (29, 30).

Our single-cell interaction analysis revealed that MSC therapy profoundly altered cell–cell communication networks, particularly those mediated by the complement system. The complement component C3 and its receptors, CD11b (Itgam) and CD18 (Itgb2), were prominently enriched within the reprogrammed MoMF compartment following MSC treatment. The identified C3-Itgam/Itgb2 and C3-Itgax/Itgb2 ligand-receptor pairs were enriched in MoMF-to-MDSC and MoMF-to-NK cell interactions, suggesting that MSCs utilize complement signaling to rewire hepatic immune crosstalk. Complement components such as C3 have been recognized as double-edged regulators in liver inflammation. While uncontrolled activation exacerbates injury, regulated complement activity facilitates tissue regeneration and immune tolerance (31, 32). Previous studies have highlighted the C3-CD11b axis in promoting macrophage-mediated adaptive tolerance (33, 34). However, our data suggest a distinct innate-centric mechanism during hyper-acute injury where MoMF-derived complement signaling directly modulates MDSCs and suppresses cytotoxic NK cell activation. These data collectively suggest that the MSC-MoMF-complement axis represents a critical pathway orchestrating immune resolution and promoting liver regeneration. Previous preclinical studies have confirmed that MSCs attenuate liver fibrosis, autoimmune hepatitis, and drug-induced liver injury through immunomodulation (10, 24, 28). However, most of these studies relied on bulk tissue analyses that failed to resolve cellular heterogeneity. Our single-cell transcriptomic approach provides a more refined understanding of how MSCs reshape hepatic immunity at the cellular and molecular levels. Specifically, the identification of MoMF-derived complement signaling toward MDSCs and NK cells provides a novel cellular basis for the durable anti-inflammatory effects observed following MSC therapy. Clinically, MSC-based therapies have shown promising results in early-phase trials for liver cirrhosis and autoimmune hepatitis, improving liver function without significant adverse effects (35–37). The mechanisms elucidated in our study provide a mechanistic foundation for optimizing MSC-based treatment protocols, including dosing, timing, and potential combination with targeted immunotherapies.

## 5 Conclusions

In summary, our study provides a comprehensive single-cell landscape illustrating how MSC therapy reprograms hepatic immune responses in ConA-induced acute hepatitis. MSCs alleviate liver injury by suppressing inflammatory cytokine production, rebalancing immune cell composition, and activating the C3-CD11b/CD18 complement signaling axis to mediate intercellular immune regulation. These findings highlight a mechanistic framework wherein MSCs coordinate macrophage-dependent complement signaling to resolve hepatic inflammation and promote tissue regeneration, offering new insights into the therapeutic potential of MSCs for immune-mediated liver diseases.

## Data Availability

The datasets presented in this study can be found in online repositories. The names of the repository/repositories and accession number(s) can be found below: https://ngdc.cncb.ac.cn/search/specific?db=bioproject&q=PRJCA050341, PRJCA050341.

## Supplementary material

Supplementary Figure 1 Serum cytokine levels in the normal control (NC), mesenchymal stem cell–treated control (MSC), Concanavalin A–challenged (ConA), and combined mesenchymal stem cell–treated ConA (ConA+MSC) groups.**Supplementary Figure 2**. Quality control metrics and global transcriptional landscape of single-cell RNA sequencing datasets. **(A)** Bar plot showing the number of high-quality cells obtained across all biological replicates from NC, MSC, ConA, and ConA+MSC groups after quality control filtering. **(B)** Violin plots displaying the distribution of total RNA counts (nCount_RNA), detected genes per cell (nFeature_RNA), and mitochondrial gene percentages (percent.mt), confirming comparable cell quality and sequencing depth among samples. **(C)** Uniform Manifold Approximation and Projection (UMAP) visualization of cells from each replicate and treatment group, demonstrating consistent clustering patterns and transcriptional identities across experimental conditions.

## References

[1] Erhardt A Biburger M Papadopoulos T Tiegs G . Il-10, regulatory t cells, and kupffer cells mediate tolerance in concanavalin a-induced liver injury in mice. Hepatology. (2007) 45:475–85. doi: 10.1002/hep.21498. PMID: 17256743

[2] Tiegs G Hentschel J Wendel A . A t cell-dependent experimental liver injury in mice inducible by concanavalin a. J Clin Invest. (1992) 90:196–203. doi: 10.1172/JCI115836. PMID: 1634608 PMC443081

[3] Kusters S Gantner F Kunstle G Tiegs G . Interferon gamma plays a critical role in t cell-dependent liver injury in mice initiated by concanavalin a. Gastroenterology. (1996) 111:462–71. doi: 10.1053/gast.1996.v111.pm8690213. PMID: 8690213

[4] Gantner F Leist M Lohse AW Germann PG Tiegs G . Concanavalin a-induced t-cell-mediated hepatic injury in mice: the role of tumor necrosis factor. Hepatology. (1995) 21:190–8. doi: 10.1016/0270-9139(95)90428-x. PMID: 7806154

[5] Sebode M Hartl J Vergani D Lohse AW International Autoimmune Hepatitis G . Autoimmune hepatitis: from current knowledge and clinical practice to future research agenda. Liver Int. (2018) 38:15–22. doi: 10.1111/liv.13458. PMID: 28432836

[6] Wang Y Chen X Cao W Shi Y . Plasticity of mesenchymal stem cells in immunomodulation: pathological and therapeutic implications. Nat Immunol. (2014) 15:1009–16. doi: 10.1038/ni.3002. PMID: 25329189

[7] Shi Y Wang Y Li Q Liu K Hou J Shao C . Immunoregulatory mechanisms of mesenchymal stem and stromal cells in inflammatory diseases. Nat Rev Nephrol. (2018) 14:493–507. doi: 10.1038/s41581-018-0023-5. PMID: 29895977

[8] Han X Liao R Li X Zhang C Huo S Qin L . Mesenchymal stem cells in treating human diseases: molecular mechanisms and clinical studies. Signal Transduct Target Ther. (2025) 10:262. doi: 10.1038/s41392-025-02313-9. PMID: 40841367 PMC12371117

[9] Liu Q Chen X Liu C Pan L Kang X Li Y . Mesenchymal stem cells alleviate experimental immune-mediated liver injury via chitinase 3-like protein 1-mediated t cell suppression. Cell Death Dis. (2021) 12:240. doi: 10.1038/s41419-021-03524-y. PMID: 33664231 PMC7933182

[10] Gao F Chiu SM Motan DA Zhang Z Chen L Ji HL . Mesenchymal stem cells and immunomodulation: current status and future prospects. Cell Death Dis. (2016) 7:e2062. doi: 10.1038/cddis.2015.327. PMID: 26794657 PMC4816164

[11] Lu D Jiao X Jiang W Yang L Gong Q Wang X . Mesenchymal stem cells influence monocyte/macrophage phenotype: regulatory mode and potential clinical applications. BioMed Pharmacother. (2023) 165:115042. doi: 10.1016/j.biopha.2023.115042. PMID: 37379639

[12] Stuart T Butler A Hoffman P Hafemeister C Papalexi E Mauck WM . Comprehensive integration of single-cell data. Cell. (2019) 177:1888–902:e21. doi: 10.1016/j.cell.2019.05.031. PMID: 31178118 PMC6687398

[13] Zheng GX Terry JM Belgrader P Ryvkin P Bent ZW Wilson R . Massively parallel digital transcriptional profiling of single cells. Nat Commun. (2017) 8:14049. doi: 10.1038/ncomms14049. PMID: 28091601 PMC5241818

[14] Ramachandran P Dobie R Wilson-Kanamori JR Dora EF Henderson BEP Luu NT . Resolving the fibrotic niche of human liver cirrhosis at single-cell level. Nature. (2019) 575:512–8. doi: 10.1038/s41586-019-1631-3. PMID: 31597160 PMC6876711

[15] MacParland SA Liu JC Ma XZ Innes BT Bartczak AM Gage BK . Single cell rna sequencing of human liver reveals distinct intrahepatic macrophage populations. Nat Commun. (2018) 9:4383. doi: 10.1038/s41467-018-06318-7. PMID: 30348985 PMC6197289

[16] McGinnis CS Murrow LM Gartner ZJ . Doubletfinder: doublet detection in single-cell rna sequencing data using artificial nearest neighbors. Cell Syst. (2019) 8:329–37:e4. doi: 10.1016/j.cels.2019.03.003. PMID: 30954475 PMC6853612

[17] Yang S Corbett SE Koga Y Wang Z Johnson WE Yajima M . Decontamination of ambient rna in single-cell rna-seq with decontx. Genome Biol. (2020) 21:57. doi: 10.1186/s13059-020-1950-6. PMID: 32138770 PMC7059395

[18] Qiu X Mao Q Tang Y Wang L Chawla R Pliner HA . Reversed graph embedding resolves complex single-cell trajectories. Nat Methods. (2017) 14:979–82. doi: 10.1038/nmeth.4402. PMID: 28825705 PMC5764547

[19] Yu G Wang LG Han Y He QY . Clusterprofiler: an r package for comparing biological themes among gene clusters. OMICS. (2012) 16:284–7. doi: 10.1089/omi.2011.0118. PMID: 22455463 PMC3339379

[20] Jin S Plikus MV Nie Q . Cellchat for systematic analysis of cell-cell communication from single-cell transcriptomics. Nat Protoc. (2025) 20:180–219. doi: 10.1038/s41596-024-01045-4. PMID: 39289562

[21] Pan L Liu C Liu Q Li Y Du C Kang X . Human wharton's jelly-derived mesenchymal stem cells alleviate concanavalin a-induced fulminant hepatitis by repressing nf-kappab signaling and glycolysis. Stem Cell Res Ther. (2021) 12:496. doi: 10.1186/s13287-021-02560-x. PMID: 34503553 PMC8427901

[22] Huang WC Li YC Chen PX Ma KS Wang LT . Mesenchymal stem cell therapy as a game-changer in liver diseases: review of current clinical trials. Stem Cell Res Ther. (2025) 16:3. doi: 10.1186/s13287-024-04127-y. PMID: 39762946 PMC11705688

[23] Luz-Crawford P Hernandez J Djouad F Luque-Campos N Caicedo A Carrere-Kremer S . Mesenchymal stem cell repression of th17 cells is triggered by mitochondrial transfer. Stem Cell Res Ther. (2019) 10:232. doi: 10.1186/s13287-019-1307-9. PMID: 31370879 PMC6676586

[24] Chen X Wei Q Sun H Zhang X Yang C Tao Y . Exosomes derived from human umbilical cord mesenchymal stem cells regulate macrophage polarization to attenuate systemic lupus erythematosus-associated diffuse alveolar hemorrhage in mice. Int J Stem Cells. (2021) 14:331–40. doi: 10.15283/ijsc20156. PMID: 33906978 PMC8429939

[25] Xiong X Kuang H Ansari S Liu T Gong J Wang S . Landscape of intercellular crosstalk in healthy and nash liver revealed by single-cell secretome gene analysis. Mol Cell. (2019) 75:644–60:e5. doi: 10.1016/j.molcel.2019.07.028. PMID: 31398325 PMC7262680

[26] Lee KC Lin HC Huang YH Hung SC . Allo-transplantation of mesenchymal stem cells attenuates hepatic injury through il1ra dependent macrophage switch in a mouse model of liver disease. J Hepatol. (2015) 63:1405–12. doi: 10.1016/j.jhep.2015.07.035. PMID: 26276675

[27] Cutolo M Soldano S Smith V Gotelli E Hysa E . Dynamic macrophage phenotypes in autoimmune and inflammatory rheumatic diseases. Nat Rev Rheumatol. (2025) 21:546–65. doi: 10.1038/s41584-025-01279-w. PMID: 40721670

[28] Feng X Feng B Zhou J Yang J Pan Q Yu J . Mesenchymal stem cells alleviate mouse liver fibrosis by inhibiting pathogenic function of intrahepatic b cells. Hepatology. (2025) 81:1211–27. doi: 10.1097/HEP.0000000000000831. PMID: 38546278 PMC11902620

[29] Gieseck RL 3rd Wilson MS Wynn TA . Type 2 immunity in tissue repair and fibrosis. Nat Rev Immunol. (2018) 18:62–76. doi: 10.1038/nri.2017.90. PMID: 28853443

[30] Tacke F Zimmermann HW . Macrophage heterogeneity in liver injury and fibrosis. J Hepatol. (2014) 60:1090–6. doi: 10.1016/j.jhep.2013.12.025. PMID: 24412603

[31] Ricklin D Reis ES Lambris JD . Complement in disease: a defence system turning offensive. Nat Rev Nephrol. (2016) 12:383–401. doi: 10.1038/nrneph.2016.70. PMID: 27211870 PMC4974115

[32] Kolev M Le Friec G Kemper C . Complement--tapping into new sites and effector systems. Nat Rev Immunol. (2014) 14:811–20. doi: 10.1038/nri3761. PMID: 25394942

[33] Bohlson SS O'Conner SD Hulsebus HJ Ho MM Fraser DA . Complement, c1q, and c1q-related molecules regulate macrophage polarization. Front Immunol. (2014) 5:402. doi: 10.3389/fimmu.2014.00402. PMID: 25191325 PMC4139736

[34] Guo RF Ward PA . Role of c5a in inflammatory responses. Annu Rev Immunol. (2005) 23:821–52. doi: 10.1146/annurev.immunol.23.021704.115835. PMID: 15771587

[35] Lin BL Chen JF Qiu WH Wang KW Xie DY Chen XY . Allogeneic bone marrow-derived mesenchymal stromal cells for hepatitis b virus-related acute-on-chronic liver failure: a randomized controlled trial. Hepatology. (2017) 66:209–19. doi: 10.1002/hep.29189. PMID: 28370357

[36] Liang J Zhang H Hua B Wang H Lu L Shi S . Allogenic mesenchymal stem cells transplantation in refractory systemic lupus erythematosus: a pilot clinical study. Ann Rheum Dis. (2010) 69:1423–9. doi: 10.1136/ard.2009.123463. PMID: 20650877

[37] Shi M Liu Z Wang Y Xu R Sun Y Zhang M . A pilot study of mesenchymal stem cell therapy for acute liver allograft rejection. Stem Cells Transl Med. (2017) 6:2053–61. doi: 10.1002/sctm.17-0134. PMID: 29178564 PMC5702514

