# Supplementary material for: Mesenchymal stem cell alleviate concanavalin A-induced hepatitis via immune reprogramming and complement regulation
Source: Front Immunol. 2026 May 13;17:1809194. doi: 10.3389/fimmu.2026.1809194 (PMC13212188; doi:10.3389/fimmu.2026.1809194)
Supplement: Supplementary Figure 1 — Serum cytokine levels in the normal control (NC), mesenchymal stem cell–treated control (MSC), Concanavalin A–challenged (ConA), and combined mesenchymal stem cell–treated ConA (ConA+MSC) groups.Supplementary Figure 2. Quality control metrics and global transcriptional landscape of single-cell RNA sequencing datasets. (A) Bar plot showing the number of high-quality cells obtained across all biological replicates from NC, MSC, ConA, and ConA+MSC groups after quality control filtering. (B) Violin plots displaying the distribution of total RNA counts (nCount_RNA), detected genes per cell (nFeature_RNA), and mitochondrial gene percentages (percent.mt), confirming comparable cell quality and sequencing depth among samples. (C) Uniform Manifold Approximation and Projection (UMAP) visualization of cells from each replicate and treatment group, demonstrating consistent clustering patterns and transcriptional identities across experimental conditions. [file Image1.pdf]

Supplementary Material

1 Supplementary Figures and Tables

1.1 Supplementary Figures

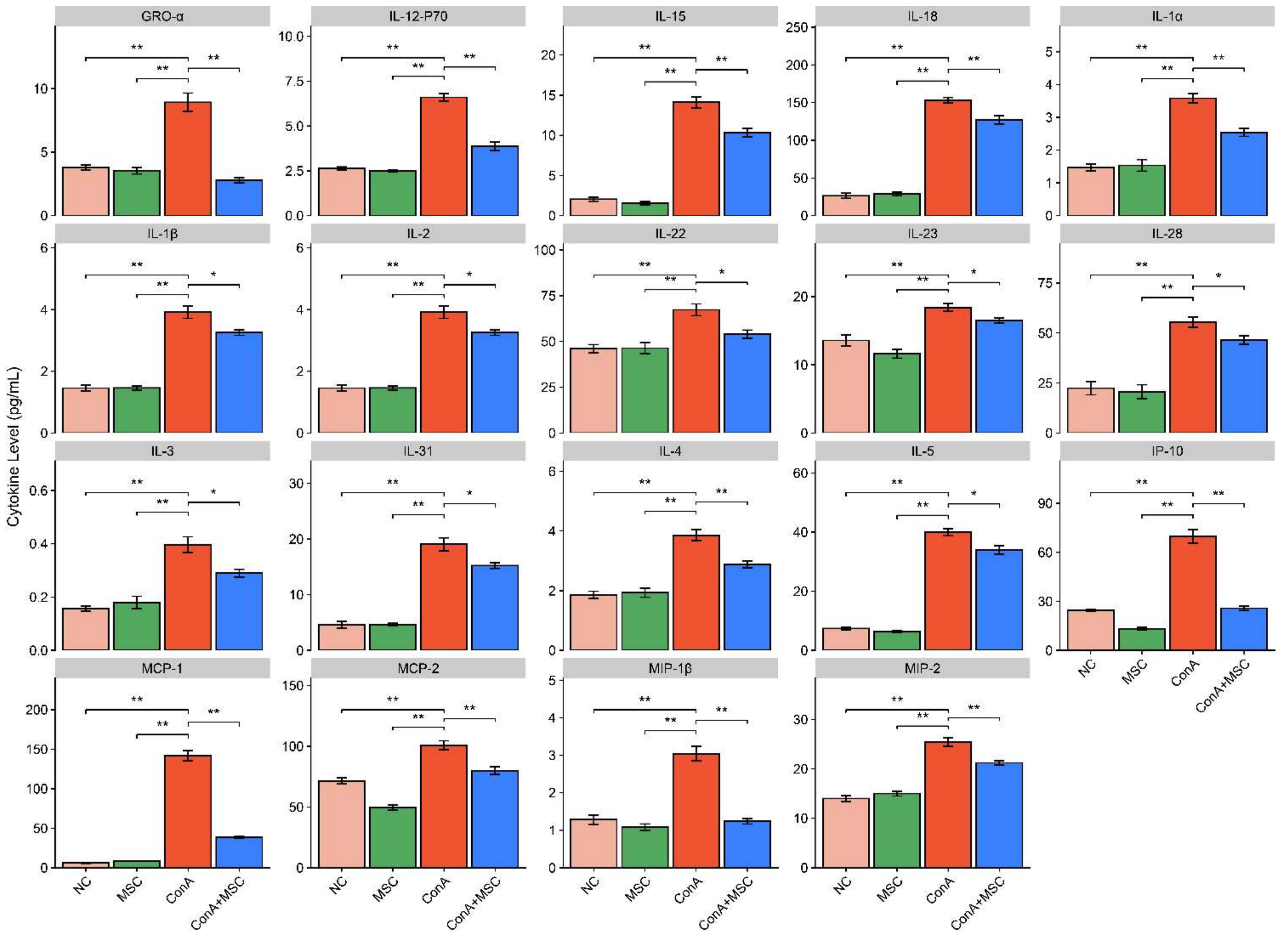

Supplementary Figure 1. Serum cytokine levels in the normal control (NC), mesenchymal stem cell–treated control (MSC), Concanavalin A–challenged (ConA), and combined mesenchymal stem cell–treated ConA (ConA+MSC) groups.

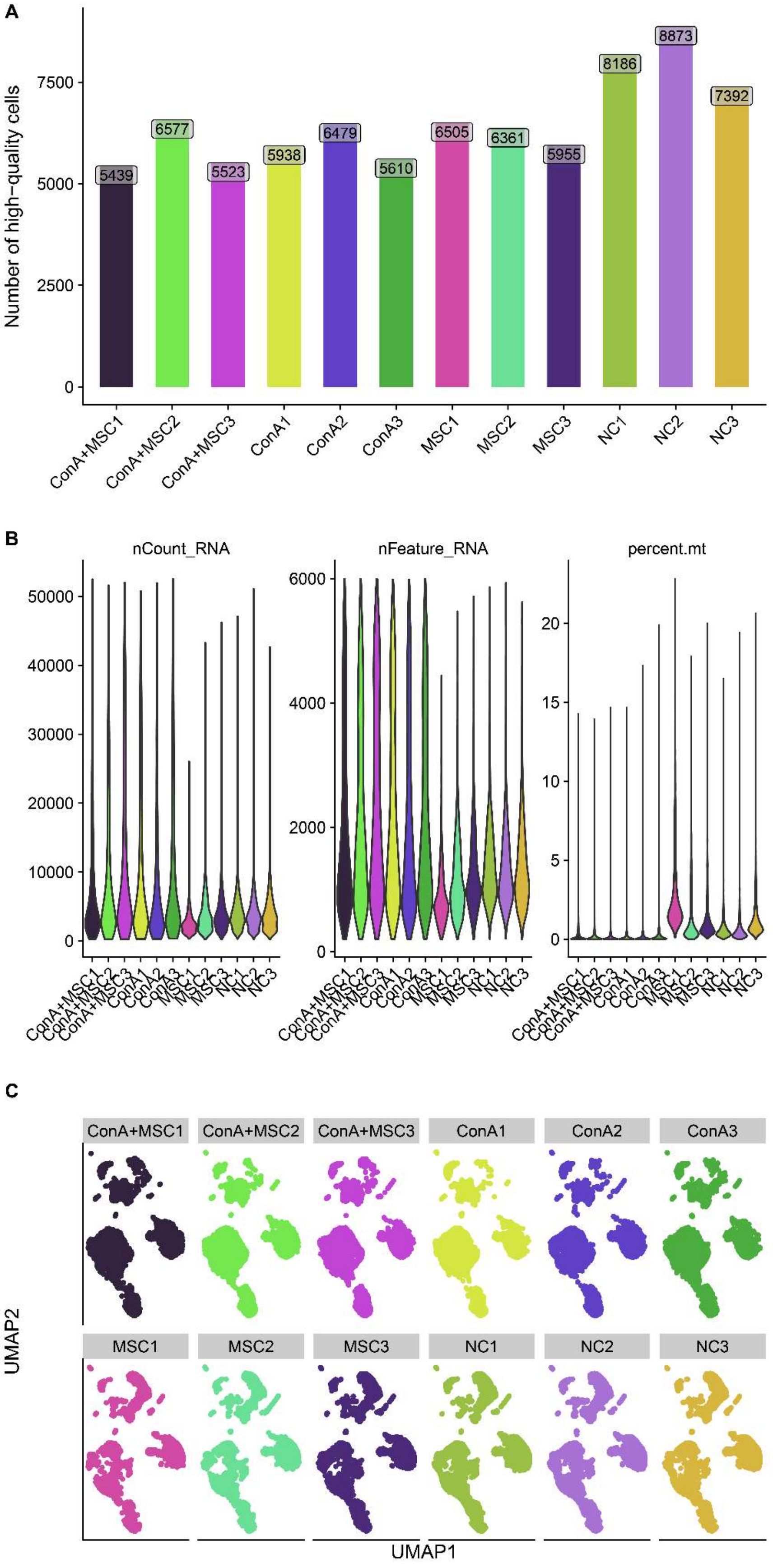
A
Number of high−quality cells
0
2500
5000
7500
5439
6577
5523
5938
6479
5610
6505
6361
5955
8186
8873
7392
ConA+MSC1
ConA+MSC2
ConA+MSC3
ConA1
ConA2
ConA3
MSC1
MSC2
MSC3
NC1
NC2
NC3
B
nCount_RNA
nFeature_RNA
percent.mt
0
10000
20000
30000
40000
50000
0
2000
4000
6000
0
5
10
15
20
C
ConA+MSC1
ConA+MSC2
ConA+MSC3
ConA1
ConA2
ConA3
MSC1
MSC2
MSC3
NC1
NC2
NC3
UMAP2
UMAP1

Supplementary Figure 2. Quality control metrics and global transcriptional landscape of single-cell RNA sequencing datasets.

(A) Bar plot showing the number of high-quality cells obtained across all biological replicates from NC, MSC, ConA, and ConA+MSC groups after quality control filtering. **(B)** Violin plots displaying the distribution of total RNA counts (nCount_RNA), detected genes per cell (nFeature_RNA), and mitochondrial gene percentages (percent.mt), confirming comparable cell quality and sequencing depth among samples. **(C)** Uniform Manifold Approximation and Projection (UMAP) visualization of cells from each replicate and treatment group, demonstrating consistent clustering patterns and transcriptional identities across experimental conditions.

2.2 Supplementary Tables

Supplementary Table 1. Differentially expressed genes (DEGs) of monocyte-derived macrophages (MoMFs) across different experimental groups.

Supplementary Table 2. Differentially expressed genes (DEGs) of the four monocyte-derived macrophage (MoMF) subclusters.
